# Social activities in multidomain dementia prevention interventions: insights from practice and a blueprint for the future

**DOI:** 10.3389/fpsyt.2024.1386688

**Published:** 2024-05-20

**Authors:** Jeroen Bruinsma, Leonie N. C. Visser, Alara Abaci, Anna Rosenberg, Ana Diaz, Sten Hanke, Rik Crutzen, Francesca Mangialasche, Miia Kivipelto, Charlotta Thunborg

**Affiliations:** ^1^Department of Health Promotion, Care and Public Health Research Institute, Maastricht University, Maastricht, Netherlands; ^2^Department of Medical Psychology, Amsterdam UMC, University of Amsterdam, Amsterdam, Netherlands; ^3^Public Health Research Institute, Quality of Care/Personalized Medicine, Amsterdam, Netherlands; ^4^Alzheimer Center Amsterdam, Department of Neurology, Amsterdam University Medical Center (UMC), Amsterdam, Netherlands; ^5^Division of Clinical Geriatrics, Center for Alzheimer Research, Department of Neurobiology, Care Sciences and Society, Karolinska Institutet, Stockholm, Sweden; ^6^Population Health Unit, Finnish Institute for Health and Welfare, Helsinki, Finland; ^7^Alzheimer Europe, Senningerberg, Luxembourg; ^8^Department of Applied Informatics, Institute of eHealth, FH Joanneum - University of Applied Sciences, Graz, Austria; ^9^Theme Inflammation and Aging, Medical Unit Aging, Karolinska University Hospital, Stockholm, Sweden; ^10^Institute of Public Health and Clinical Nutrition, University of Eastern Finland, Kuopio, Finland; ^11^The Ageing Epidemiology Research Unit, School of Public Health, Imperial College London, London, United Kingdom; ^12^Department of Caring Sciences, Faculty of Health and Occupational Studies, University of Gävle, Gävle, Sweden

**Keywords:** social activities, prevention, cognitive decline, dementia, multidomain intervention

## Abstract

**Introduction:**

Social activities are important for health and act as a driver of cognitive reserve during aging. In this perspective paper, we describe challenges and outline future (research) endeavors to establish better operationalization of social activities in multidomain interventions to prevent dementia.

**Body:**

We first address the lack of conceptual clarity, which makes it difficult to measure engagement in social activities. Second, drawing from our experience with the Finnish Geriatric Intervention Study to Prevent Cognitive Impairment and Disability (FINGER), we discuss social activities in multidomain dementia prevention interventions. Using qualitative data from the Multimodal Preventive Trial for Alzheimer’s Disease (MIND-AD_mini_), we reflect on participant experiences with social activities. Third, we address the potential and challenges of digital solutions in promoting social activities in interventions for dementia prevention. Finally, we share insights from a workshop on digital technology, where we consulted with individuals with and without cognitive impairment who have been involved in three European projects (i.e., EU-FINGERS, Multi-MeMo, and LETHE).

**Discussion:**

Based on these insights, we advocate for research that strengthens and accelerates the integration of social activities into multidomain interventions for dementia prevention. We propose several ways to achieve this: (a) by conducting mixed methods research to formulate a broadly accepted definition and instructions to measure social activities; (b) by focusing on promoting engagement in social activities beyond the intervention setting; and (c) by exploring the needs and preferences of older adults towards digitally-supported interventions and co-design of new technologies that enrich in-person social activities.

## Introduction

Discovering ways to prevent dementia or slow down the progression of underlying diseases such as Alzheimer’s disease (AD) has a high priority world-wide. According to the 2020 Lancet Commission, tackling 12 identified modifiable risk factors could potentially prevent around 40 percent of dementia cases globally. Social isolation is one of the risk factors that contributes 3.5% of the overall 39.7% of the population attributable fraction of dementia worldwide (1). Social isolation is characterized by an individual’s lack of meaningful interactions (2), and is related to other risk factors for dementia, such as depression (1). This indicates that social isolation and other risk factors can exacerbate each other, which stresses its importance. During the COVID-19 pandemic, social isolation has become more apparent, especially among older adults. This possibly results in negative cognitive health outcomes (3, 4), since social activities are hypothesized to contribute to cognitive reserve (5) and older adults who are socially active tend to experience less cognitive decline as they age (6–13). Yet, methodological issues, including a lack of robust cognitive assessment, reverse causation, and confounding factors, are suggested as alternative explanations for finding protective effects of social activities against dementia (13). In 2019, the World Health Organization (WHO) concluded that there is currently insufficient evidence to recommend social activity as a strategy to reduce dementia risk (14). More recently, Lenart-Bugla et al. (15) reviewed evidence from systematic reviews and conclude that social support and interactions seem to protect against cognitive decline, but the evidence is inconsistent. Among the challenges is that the etiology of late-life cognitive decline is complex and multifactorial, given the lifelong cumulative exposure to multiple risk and protective factors, which means there is a need to address several risk factors simultaneously (16). Protective factors, including physical, cognitive, and social factors, as well as education received during childhood and early adulthood, play an important role in preserving cognitive health (16). Due to insufficient evidence and the challenge of addressing multiple factors simultaneously, both research and evidence about the cognitive benefits of social activities is limited and scattered (14). Despite the lack of conclusive evidence, both the United Nations’ Decade of Healthy Ageing (2021-2030) (17), and the WHO have recently recognized social health as a priority for the upcoming years (i.e., 2024-2026) (18).

Many lifestyle behaviors, such as exercising or engaging in cognitive activities, can incorporate a social dimension. As a result, it is challenging to distinguish social activities from other activities (1). Additionally, social activities are often indirectly promoted through other activities, like group exercise sessions. In this perspective paper, we argue for a dedicated focus on promoting social activities within multidomain dementia prevention interventions. Therefore, we first address the complexity of defining ‘engagement in social activities’ which contributes to the ambiguity around the operationalization in measurements and interventions. Secondly, we reflect on social activities that are incorporated into existing multidomain interventions that offer a targeted prevention approach for older adults with risk factors for dementia. Drawing on our experience with the Finnish Geriatric Intervention Study to Prevent Cognitive Impairment and Disability (FINGER) (19–22) and the Multimodal Preventive Trial for AD (MIND-AD_mini_) (23), we explore how social activities are integrated into these interventions and experienced by participants. Thirdly, we explore the potential and challenges of using digital technology in the context of these multidomain interventions, especially for monitoring and promoting social activities. Finally, we share insights from a workshop on digital technology that was organized by Alzheimer Europe and allowed us to consult members of the public, including individuals living with cognitive impairment or dementia. In the discussion, we propose directions for the future to strengthen and accelerate the integration of social activities in research and interventions aimed at dementia prevention.

## The concept of engagement in social activities: definition and measurement

Social health includes the capacity to fulfill potential and obligations, maintaining independent despite medical conditions, and engagement in social activities (24). The term ‘social activity’ is however not defined in the Merriam-Webster Dictionary, nevertheless ‘activity’ is stated as a behavior or action while ‘social’ refers to pleasant companionship with friends or associates (25). This indicates that engagement in social activities concerns a behavior performed in the company of others. In literature, many related and overlapping terms are used interchangeably to describe engagement in social activities, such as social participation, social connectedness, social cohesion, social support, social network, social integration, and community involvement (26). Moreover, social isolation – as indicated by a lack of social contact – and loneliness – a sense of feeling alone – are concepts that are closely related to the engagement in social activities. Many of these distinct yet related concepts collectively impact the domain of social health, which can positively contribute to the preservation of cognitive abilities when aging (5). The operationalization of social health in research often overlooks this multidimensionality and is typically focused on the measurement of isolated aspects (27). Compared to social isolation and loneliness, the concept of engagement in social activities is particularly vaguely defined, with few validated questionnaires available for its assessment (28). Conceptual clarity is an inaugural step towards the development and validation of effective measurements (29, 30). Presently, the absence of a clear, broadly accepted definition poses challenges to operationalize an appropriate questionnaire to capture the engagement in social activities (31, 32).

In current research that explores risk factors for dementia, the operationalization of engagement in social activities often relies on questionnaires assessing frequency and duration of activities, such as going out, socializing, volunteering, and participation in clubs and communities (28). The engagement in social activities is complex to measure, as it should ideally mirror the diverse experiences of everyday life (31, 33). Various dimensions seem relevant (31), ranging from small and sporadic encounters with acquaintances (34) to in-depth emotional interactions with close relatives or friends (10). Potentially, measurements should evaluate the physical and subjective context of social engagements, rather than relying solely on frequency and duration (35). For instance, by assessing what activities are involved, who participates, where they take place, when they occur, and whether and why they are meaningful (33). Additionally, evaluations of expectations, perceived value, and satisfaction derived from activities would provide useful insights.

Across studies there is a considerable variety in which social activities are assessed (28). Additionally, engagement in social activities is frequently evaluated alongside the engagement in cognitive activities, such as reading, gaming, puzzling (36, 37). Typically, item scores are merged into a single composite score (38, 39), which overlooks the separate and distinct influence of social activities. Additionally, a high level of heterogeneity is observed across studies in terms of the thematic content of items, response scales utilized, and the recall periods specified (28). These inconsistencies make it difficult to compare findings across studies, which results in fragmented evidence about the protective effects of social activities on dementia risk. Comprehensive, validated measurements are urgently needed to align methods across trials and other research. This allows harmonization of data, which is needed to obtain a deeper understanding in the effects of social activities against dementia (14). This knowledge could result in more nuanced risk profiles, which help to tailor the content of future multidomain interventions to the individual.

## Social activities in FINGER-model interventions for dementia prevention

Randomized controlled trials (RCTs) have provided promising evidence for the beneficial effects of multidomain interventions in older adults, to reduce the risk of cognitive decline (40). The FINGER-model interventions represent a form of targeted prevention directed at individuals on the at-risk continuum for dementia. This includes individuals with risk factors but without symptoms or brain damage, as well as those who are asymptomatic or early-symptomatic and have brain pathology (41). Regarding social activities, these multidomain interventions often include both individual and group activities (42), for instance group exercising and cognitive training (43). In the FINGER trial, the 2-year multidomain intervention included nutritional guidance, exercising, cognitive training, social activities, and management of metabolic and vascular risks, whereas a control group received general health advice. After two years, the multidomain intervention showed beneficial effects on cognition and quality of life as well as reduced risks of functional decline, cardiovascular accidents, and multimorbidity (22). In the FINGER trial, engagement in social activities between study participants was stimulated through group sessions regarding nutritional guidance, exercising, and psychoeducation about cognition and memory (23). Approximately 30% of the intervention participants self-reported an increase in cognitive and social activities. These participants exhibited slightly more improvement in their cognitive functioning (21). Moreover, 80% of the participants enjoyed meeting others involved in the intervention (44). This enjoyment may enhance intervention adherence, which is pivotal for the success of establishing lifestyle change.

The positive findings of the FINGER trial led to the World-Wide FINGERS (WW-FINGERS) global network of multidomain intervention trials for dementia risk reduction (45). Through this network over 60 countries are testing locally adapted versions in different cultural settings to evaluate feasibility and efficacy. These multidomain interventions target the whole spectrum of dementia risk, including persons with risk factors who are cognitively healthy, have mild cognitive impairment, or prodromal AD. One example is the MIND-AD_mini_ RCT, that tested the feasibility of a FINGER-based multidomain intervention, alone or with medical food, in individuals with prodromal AD (23). Participants clearly perceived benefits from the social activities, including forming friendships, laughing about jokes, sharing personal stories, discussing experiences related to their diagnoses and daily life. These activities fostered a sense of mutual understanding, which was experienced as important by participants with cognitive symptoms.

“We are a good group of friends, or have become, I think, in every way. We have fun and laugh, and we each do our best in every possible way.”

“I also feel the companionship with those in the group, that have the same illness or whatever it is called. We talk openly about it, compare a bit with each other, and can laugh about it and get serious about it, and yes, it’s great that we’re a group with exactly the same thing.”

Participants experienced social support through collaborative activities. This was evident in actions such as offering encouraging cheers, waiting for each other after group sessions, and adapting the intensity of exercises to the group’s needs. The group setting also allowed to socialize during sessions, which participants perceived as a benefit compared to exercising alone as it created a sense of belonging. These meaningful, interpersonal dynamics were perceived as crucial for adherence and continuation of intervention participation.

“Yes, but if there’s something we do, it’s one thing at a time. I do or you do, we say, and the rest of us stand there watching and say, ‘Come on now, come on now, one more time,’ or something like that. That’s how it often goes.”

“We are a group, we know a lot about each other, we have coffee afterward, we support each other.”

## Digital technologies to promote social activities in dementia prevention interventions

Digital technologies and tools, including wearables, sensors, and mobile phones, can improve the effectiveness and quality of healthcare, also in the domain of AD and dementia (46), for example by detecting and monitoring change in cognitive functioning. This could support timely identification of individuals in at-risk or in early-disease stages and improve the delivery of preventive interventions (47). For instance, digitally-supported interventions can be personalized and offer the flexibility to participate at convenient times and places, thereby making these interventions more accessible, even for participants in geographically isolated areas. Digital interventions also have the potential to be scalable, widely accessible, and relatively low in costs (48). This is particularly relevant given that the ageing population leads to an increase in healthcare demand and simultaneously results in a shrinking (healthcare) workforce (49, 50). The implementation of digital technologies offers opportunities to improve the delivery of preventive interventions. Digital tools can alleviate the workload on healthcare professionals, allowing them to dedicate more personal support to individuals who struggle with technology (51). It is crucial that technology does not seek to replace personal interactions, and that careful consideration is given to digital inequality to ensure that preventive interventions are accessible to those who need them most (1).

Early dementia lifestyle-based preventive interventions predominantly relied on trained professionals to deliver the intervention, and social interaction was stimulated by bringing participants together (40). The recent generation of trials often adopt a fully-digital or digitally-supported design, such as studies on the Healthy Aging Through Internet Counselling in the Elderly (HATICE) (52, 53), Maintain Your Brain (54), MyCoach (55), or APPLE Tree (56). Although digital interventions enable participants to access the intervention from their own home, which could stimulate adoption and adherence, it seems counter intuitive when stimulating social activities. Still, there are behavioral change methods that transfer well to the digital context, which may be useful to stimulate social activity, including psychoeducation, modelling, and social support (57–59). For instance, in APPLE Tree online video-call ‘tea breaks’ are organized to enhance social support (56). The weak tie network theory posits that such digital interactions, which involves weaker social interactions, can be beneficial for socially stigmatized conditions like dementia (60).

The two-year multinational LETHE RCT, which is a novel study within WW-FINGERS, is an example of a digitally-supported multidomain intervention for dementia risk reduction (61, 62). In LETHE, digital tools are used to complement the in-person activities to streamline the intervention delivery, personalize recommendations, and collect digital biomarkers through a smartwatch and smartphone application. In LETHE, the feasibility of using digital tools to measure social activity is explored, since these tools allow for unobtrusive monitoring of lifestyle behavior patterns, such as step counting and sleep tracking. This approach not only helps to personalize the intervention but also generates a context-rich longitudinal dataset. While tracking certain behaviors with technology is relatively straightforward, monitoring social activities is challenging. Although digital biomarkers exist for social activity, it is uncertain how accurately they reflect social interaction. These include the time spent outdoors, number of social contacts, calendar entries, social network size, social media usage, and exchanged text messages (46). Additionally, it is crucial to acknowledge that the use of these digital biomarkers raise privacy concerns, necessitating careful reflection of whether the data collected justifies the methods used.

## A workshop on digital technologies to promote social activities

The use of digital technology in multidomain interventions for dementia prevention seems promising, but the success critically depends on the alignment of technology with the needs and preferences of the people who need to use them (63). Therefore, in the multinational EU-FINGERS, Multi-MeMo, and LETHE projects, public involvement is achieved by including members of the public (i.e., with and without cognitive impairment) through project Advisory Boards (64–66). These three Advisory Boards include 10 males and 10 females from Finland (*n* = 4), Sweden (*n* = 4), Luxembourg (*n* = 1), United Kingdom (*n* = 2), Netherlands (*n* = 2), Spain (*n* = 1), Hungary (*n* = 2), Austria (*n* = 2), Italy (*n* = 2). The boards include individuals at risk (*n* = 7), with cognitive complaints (*n* = 9), with dementia (*n* = 2), and caregivers (*n* = 2). Their ages range from 45 to 70 years, with most in their 60s.

**Figure 1:**
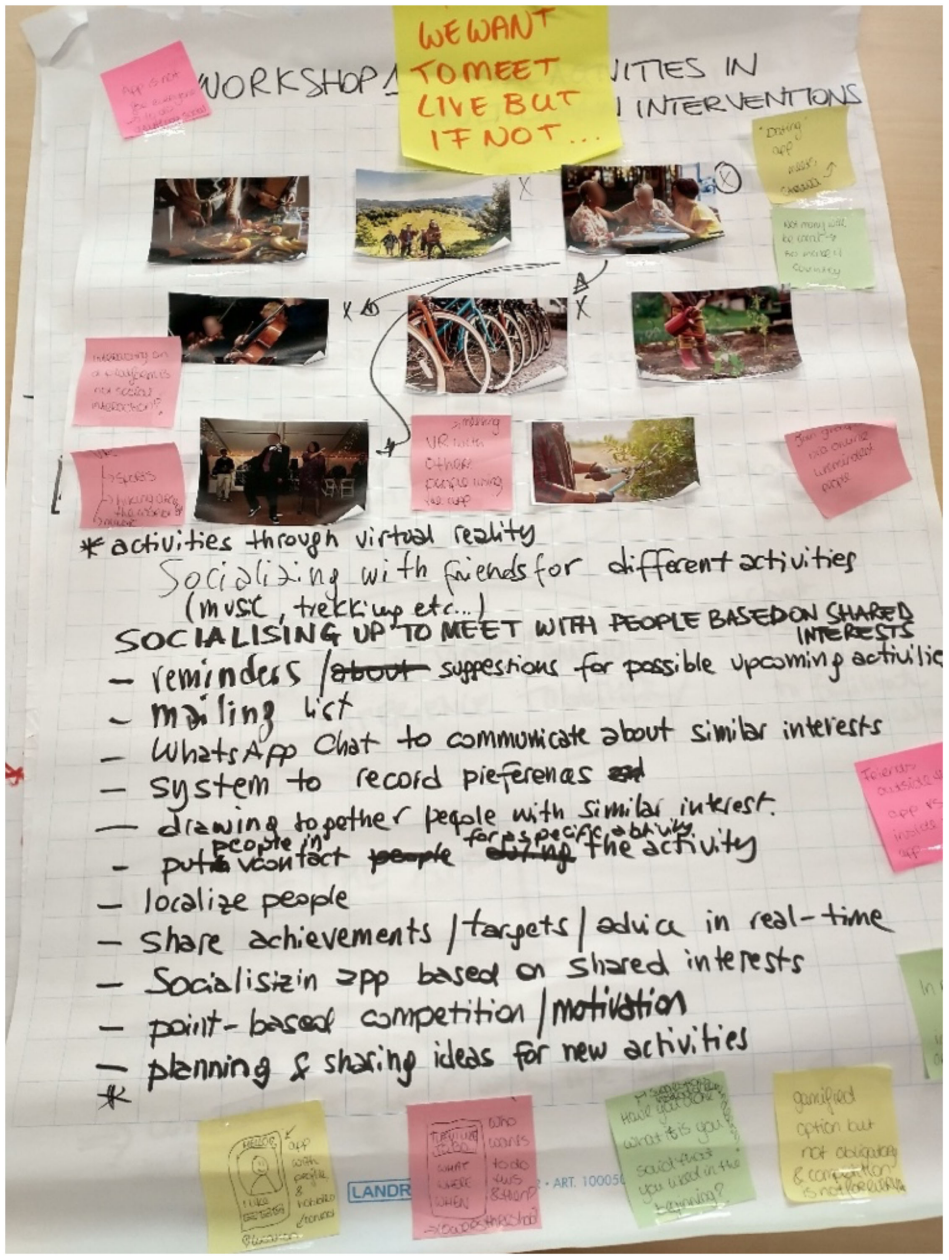
An example of output from the workshop.

In November 2023, Alzheimer Europe hosted a face-to-face workshop attended by 17 members of these Advisory Boards, where they brainstormed in four smaller subgroups about the use of digital tools to stimulate social activities (see **Figure 1** for a snippet of the output). Based on their written input and our fieldnotes, we synthesized four take away messages relevant for the future development of technology-supported multidomain dementia prevention interventions and social activities. First, according to members of the Advisory Boards social activities do not occur in isolation. They highlighted that most social activities intersect with other lifestyle domains and should be stimulated simultaneously. For example, through group exercising, playing board games, or cooking. Second, they strongly preferred meeting in person and mentioned that digital technology could help to connect with others who have similar interests. They envisioned digital technology could link participants who share hobbies, to organize and plan activities together, to inform others about community-based activities, or to share lifestyle progress with others. Third, members from the Advisory Boards recommended to rely on already existing and relatively simple digital tools, such as (private) WhatsApp groups, Facebook pages, or other social media and message-based services. They also brainstormed about more advanced applications such as a dating app to meet participants with shared interests and hobbies, as well as social interaction established by means of virtual reality. Lastly, they raised their concerns regarding safety, privacy, and trustworthiness. For instance, by stressing the importance of preventing that participants share too much personal details and to ensure that others cannot take advantage of the information shared.

## Discussion

The connection between engagement in social activities and cognitive health is promising for preventive endeavors (67–69). Currently, social activities in our, and many other, multidomain dementia prevention studies are mainly stimulated through group sessions (22, 70). However, there is room for improvement, by promoting engagement in social activities that occur beyond the intervention setting, and adopting appropriate behavior change strategies and digital tools. To accommodate this, we present directions for the future.

a) The considerable heterogeneity in measuring engagement in social activities poses challenges for cross-study comparison and results in fragmented insights into protective effects of these activities against dementia (28). Although diversity in assessment can be beneficial for epistemic diversity and scientific progress (30), the current practice of measuring engagement in social activities lacks clear and standardized criteria. The heterogeneity in measurements, and particularly the limited transparency about it, undermines the validity of findings and the quality of research in this area (28, 71). Partly, this seems is attributed to the lack of conceptual clarity, which leaves too much freedom for the measurement of social activities (29). This can be addressed by explicitly defining concepts and formulating assessment instructions aligned with these definitions (30). Research is underway to develop a broadly accepted definition and appropriate measurement instructions to assess engagement in social activities (28, 72). This research is guided by frameworks for developing conceptual definitions (29, 73) and includes mixed methods research to triangulate literature through an integrative review, expert opinion using think-aloud sessions, and the perspectives of older adults through conducting interviews.
b) Multidomain preventive interventions should prioritize stimulating engagement in community-based activities and everyday interpersonal interactions. Our qualitative findings reveal that the inclusion of social activities support the forming new, meaningful groups that provide social opportunities that provide social support. Beyond creating these new “within-trial” social connections through group sessions, it seems promising to also encourage social involvement in local clubs, communities, and strengthening ties with relatives and friends (10, 40). This requires a well-thought, systematically planned, and theoretically-sound intervention approach including behavior change methods targeting relevant determinants to promote engagement in social activities (74, 75). For instance, through verbal persuasion about the benefits, role modelling, guided practice or by mobilizing social support (76). To select appropriate behavior change methods, more research is needed to obtain a deeper understanding of the psychological determinants that drive engagement in social activities. The use of behavior change theories, such as the Social Cognitive Theory (77), may provide useful insights into how personal and environmental factors influence the adherence to preventive interventions. This would guide the embodiment of behavior change methods to support long lasting engagement in a wide range of social activities that extend beyond the trial duration. The large network of WW-FINGERS provides a unique opportunity to further explore the incorporation of new interventions to promote social activities. For instance, the 2-year multidomain AGELESS trial in Malaysia already experiments with recreational activities offered by local community centers (78).
c) Digital technologies present innovative opportunities for monitoring and stimulating social activities in multidomain interventions for dementia prevention (46). WW-FINGERS RCTs, like LETHE (61), incorporate qualitative interviews to explore and evaluate the needs and preferences of older adults towards digitally-supported multidomain interventions. Other WW-FINGERS RCTs have also used digital technology (55, 79), which allows to further explore the benefits in terms of intervention delivery, monitoring adherence, and efficacy. The effectiveness of digital technology depends on the alignment with older adults’ needs and preferences to ensure usability, feasibility, and acceptability. Therefore, co-design should be employed to carefully develop new technologies that aim to enrich in-person social activities for older adults, rather than replacing real-world interactions. If researchers work in partnership with older adults, it empowers both parties (80) to ensure that the technological solutions created are based on needs and preferences (81).

In conclusion, social health and engagement in social activities should be further incorporated within multidomain interventions for preventing cognitive decline and dementia. Stimulating social activities could benefit cognitive health directly and also increase adherence to interventions to generate long-lasting effects. In this perspective paper, we identified key challenges and proposed directions for future research including: (a) conducting mixed methods research to formulate a broadly accepted definition and instructions to measure social activities; (b) focusing on the promotion of engagement in social activities that occur outside the intervention setting; and (c) by exploring the needs and preferences of older adults towards digitally-supported interventions using co-design to develop new technologies that enrich in-person social activities. These research endeavors could contribute to, and accelerate, the improved integration of social activities in multidomain dementia prevention interventions.

## Data availability statement

The raw data supporting the conclusions of this article will be made available by the authors, without undue reservation.

## Ethics statement

The studies involving humans were approved by Regional Ethical Review Board (Regionala Etikprövningsnämnden) in Stockholm, Sweden (Registration number: 2016/2605-31/1). The studies were conducted in accordance with the local legislation and institutional requirements. The participants provided their written informed consent to participate in this study.

## Author contributions

JB: Conceptualization, Data curation, Formal analysis, Funding acquisition, Investigation, Methodology, Project administration, Resources, Software, Supervision, Validation, Visualization, Writing – original draft, Writing – review & editing. LV: Conceptualization, Data curation, Formal analysis, Funding acquisition, Investigation, Methodology, Project administration, Resources, Software, Supervision, Validation, Visualization, Writing – original draft, Writing – review & editing. AA: Formal analysis, Investigation, Writing – original draft, Writing – review & editing, Conceptualization. AR: Writing – original draft, Writing – review & editing. AD: Writing – original draft, Writing – review & editing. SH: Writing – original draft, Writing – review & editing. RC: Conceptualization, Writing – original draft, Writing – review & editing. FM: Writing – original draft, Writing – review & editing. MK: Conceptualization, Writing – original draft, Writing – review & editing. CT: Conceptualization, Data curation, Formal analysis, Funding acquisition, Investigation, Methodology, Project administration, Resources, Software, Supervision, Validation, Visualization, Writing – original draft, Writing – review & editing.
